# Prevalence and risk factors of Hepatitis C virus infection in the Upper East Region of Ghana; a community-based cross-sectional study

**DOI:** 10.1371/journal.pone.0306292

**Published:** 2024-06-28

**Authors:** Ampem Darko Jnr Siaw, James Amugsi, Maame Adwoa Agyeman Owusu-Konadu, Samuel Teye Drah, Emmanuel Gustav Imbeah, Dominic Oduro-Donkor, Amoako Duah, Yvonne Ayerki Nartey

**Affiliations:** 1 Department of Internal Medicine, Cape Coast Teaching Hospital, Cape Coast, Ghana; 2 Out-patient Department, Sandema Hospital, Sandema, Ghana; 3 Department of Anaesthesia and Intensive Care Unit, University of Ghana Medical Center, Accra, Ghana; 4 Department of Internal Medicine, Ledzokuku-Krowor Municipal Assembly Hospital, Accra, Ghana; 5 ACT Pathology Consult, Cape Coast, Ghana; 6 Warwickshire Institute for the Study of Diabetes, Endocrinology and Metabolism, University Hospitals Coventry and Warwickshire, Coventry, United Kingdom; 7 Department of Medicine, University of Ghana Medical Centre, Accra, Ghana; 8 Department of Internal Medicine and Therapeutics, University of Cape Coast, School of Medical Sciences, Cape Coast, Ghana; Centers for Disease Control and Prevention, UNITED STATES

## Abstract

Hepatitis C virus (HCV) infection remains a major cause of liver related morbidity and mortality worldwide. Epidemiologic data on seroprevalence, viremia prevalence and risk factors remain limited in sub-Saharan Africa. In Ghana, HCV-related deaths are estimated to have increased since 2015. Risk factors associated with HCV infection in Ghana are not well described. The aim of this study was to determine the prevalence of, and risk factors associated with hepatitis C virus infection in the Upper East Region located in the northern part of Ghana. A community-based cross-sectional study was conducted in 9 communities in the Upper East region of Ghana. A total of 1,769 participants aged ≥12 years were screened for HCV antibody (anti-HCV) using rapid diagnostic testing (RDT). Seventy-four participants undertook HCV RNA testing after a positive anti-HCV result. Multivariate logistic regression was used to determine risk factors associated with HCV seropositivity. The anti-HCV prevalence was 8.4%, with 149 out of 1,769 testing anti-HCV positive. Mean age (±SD) of seropositive persons was 45.4 (±16.3) years. The highest anti-HCV seroprevalence was amongst persons aged 60 years and above. Forty-four out of 74 (59.5%) seropositive cases had viremic infection and the estimated viremic prevalence in the screened population was 5.0%. Predictors of HCV seropositivity were age (OR 1.03 95% CI 1.01–1.04), history of female genital mutilation or circumcision (OR 1.63 95% CI 1.04–2.55), sexual activity (OR 2.57 95% CI 1.38–4.79), positive maternal HCV status (OR 10.38 95% CI 4.13–26.05) and positive HIV status (OR 4.03 95% CI 1.35–12.05). In conclusion, the Upper East Region demonstrates a high Hepatitis C antibody prevalence. Almost 60% of individuals have viremic infection, however the cost of RNA testing is a barrier to virological diagnosis. There is a need to educate the population about HCV-associated risk factors to reduce HCV transmission and burden of disease.

## Introduction

Globally, Hepatitis C virus (HCV) is one of the leading infectious causes of liver cirrhosis and hepatocellular cancer [1]. Worldwide roughly 21% of patients diagnosed with liver cirrhosis have HCV infection [2]. The World Health Organization (WHO) estimates that globally there are 58 million people living with chronic hepatitis C infection, and that each year 1.5 million new HCV infections occur [3]. In sub-Saharan Africa (SSA), roughly 9 million people are infected with HCV and the estimated seroprevalence is 2.98% with variations among African sub-regions [4]. Furthermore, HCV is the second leading cause of mortality from liver failure and hepatocellular carcinoma in SSA [5]. HCV is curable with Direct Acting Antiviral (DAA) therapy, however of the 58 million people infected globally in 2019, only one in five were aware of their diagnosis, and only 9.4 million people worldwide received treatment [3].

In 2016, the Global Health Sector Strategy (GHSS) on viral hepatitis was launched by the World Health Assembly with the goal of eliminating viral hepatitis by 2030 [6]. In the updated strategy for 2022–2030, key programmatic aims include reduction in incidence of HCV infection to 5 per 100,00 and a reduction in mortality to 2 per 100,00. The GHSS additionally aims for 90% of infected individuals to be diagnosed and 80% of individuals diagnosed to be treated by 2030 [6,7]. To track progress towards achievement of the GHSS goals, primary data on HCV infection are required. However, prevalence and incidence data related to HCV in SSA are not well described [5,8].

In Ghana, the prevalence of HCV antibody was reported at 3.0% in a systematic review comprising hospital-based and limited community-based studies in 2016 [9]. Furthermore, hospital-based data from Ghana demonstrated a crude anti-HCV prevalence of 2.6% [10], and HCV is associated with 6.4% of Hepatocellular Carcinoma (HCC) [11] cases and 7% of liver-related deaths [12]. Previous studies on HCV in Ghana have primarily relied on hospital-based data, which may limit our ability to determine prevalence in the general population, therefore there is a need for community-based studies to better characterize the burden of infection. Furthermore, there is little evidence on the viremia or chronic HCV prevalence in Ghana from population-based studies, with previous estimates based on modeled data [4]. Moreover, where earlier studies have demonstrated high seroprevalence regions of HCV within the north of Ghana [10], little is known about the risk factors associated with HCV in these regions, where there are different socio-cultural norms, high poverty rates, less access to health care and low educational levels compared with southern Ghana [13,14].

This study aims to determine the antibody and viremia prevalence, and associated risk factors of HCV infection in a community in the Upper East region located in the northern part of Ghana. The overall goal is to provide further epidemiological context, which may aid in the development and implementation of public health interventions to eliminate viral hepatitis in Ghana.

## Methods

### Study design and study setting

A community-based cross-sectional study was conducted in nine communities in Sandema, in the Builsa North District of the Upper East Region of Ghana from 1^st^ September to 30^th^ November 2022. The communities studied were Abilyire, Balansa, Chuchuliga central, Nanjopiung, Akuteiyire, Wiaga, Siniensi, and Sandema Senior High and Technical school. The Upper East region has an estimated population of about 948,118 people and Bolgatanga is the capital with an approximate distance of 543km from Accra, the capital of Ghana [15].

### Sample size and sampling method

A total of 1,769 individuals were included in the study. Using Cochran’s formula [16] with a 95% confidence level and an estimated proportion of 14.4% based on previously reported seroprevalence [10], the minimum sample size required was 190. Participants were consented based on convenience sampling, with screening offered to inhabitants of the nine communities visited as part of World Hepatitis Day 2022—related activities in the district. Screening was conducted by staff of the local district hospital including a physician assistant, nurses, and laboratory personnel who were experienced with community-based screening and interventions in the nine communities. This was a facilitating factor in recruitment since the district hospital is known to the community.

### Study population, inclusion, and exclusion criteria

Residents of the nine communities in which free anti-HCV screening was performed comprised the study population. The inclusion criteria were individuals aged 12 years or older and with weight above 35kg who provided informed consent. The age and weight cut-offs were chosen in-line with the enrollment requirements for a free HCV treatment project by the National Viral Hepatitis Control Program of the Ghana Health Service called Screening and Treatment Opportunity Project for Hepatitis C (STOP Hep C) [17,18] so that participants who underwent screening would directly benefit from this program. The STOP Hep C project does not cover the cost of testing but provides free treatment for individuals identified to have viremic HCV infection. Individuals already on HCV therapy were excluded from the study.

### Data collection

A Wondfo One Step Hepatitis C Virus Rapid Diagnostic test was used for the screening of whole blood samples from participants as per the manufacturer’s instructions. The manufacturer reports a sensitivity of the Wondfo test kit of 96.7% and specificity of 97.5% from whole blood, serum or plasma samples [19]. HCV RNA testing of antibody-positive serum samples was conducted using the GeneProof^®^ Hepatitis C Virus Diagnostic PCR Kit as per the manufacturer’s instructions. A structured questionnaire with socio-demographic information and risk factors of HCV infection was prepared after thorough review of the literature. Questionnaires were administered to study participants by trained study personnel. Data gathered were subsequently abstracted into Microsoft Excel.

### Data analysis

Descriptive statistics for age and sex were reported using mean with standard deviation and median with interquartile range for continuous variables and frequencies for categorical variables. Seroprevalence was calculated by dividing the total number of seropositive individuals by the total population screened, and viremia proportion was calculated by dividing the total number of persons who were HCV RNA positive by the total number of seropositive individuals. An estimate of viremic prevalence was calculated by multiplication of the seroprevalence by viremia proportion. The t-test was used to compare the means of continuous variables. The Chi-square test was used to compare relationships between independent groups of categorical variables. Predictors of seropositivity were determined using binary logistic regression adjusted for age, sex, and risk factors to calculate odds ratios and 95% CIs. In the logistic regression models, anti-HCV positivity was modelled as the outcome, and variables modelled as predictors included: age (continuous), sex (male/female) and risk factors (yes/no). For statistical significance, a significance level of 0.05 was used. All tests were two-sided with a 95% confidence interval (p-value 0.05). Data analysis was performed using Stata, version 17, Stata Corp software.

### Ethical considerations

The study was conducted after ethical approval was granted by the Ethics Review Committee (ERC) of the Ghana Health Service (GHS-ERC 013/06/22 and GHS-ERC 013/03/23). The study objectives, benefits and risks were explained to all participants directly or through guardians after which a thumbprint or signed written consent was obtained. Participants were made aware that all information provided was confidential. Seropositive individuals were advised on the need to undertake HCV viral load testing and were provided information on case management sites of the STOP Hep C Ghana project [17,18] for linkage to and continuity of care.

## Results

**Fig 1:**
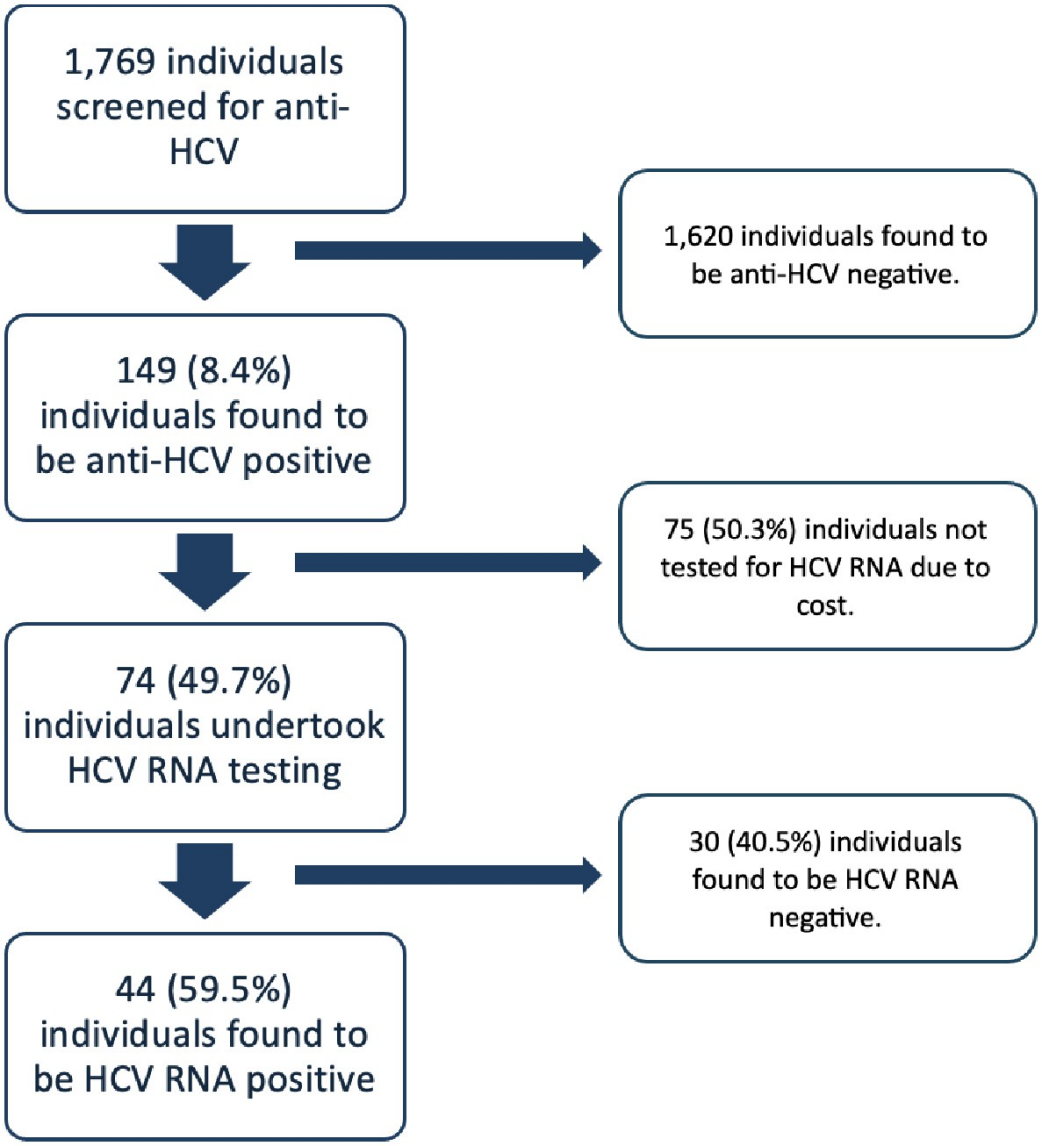
Flow diagram illustrating study procedures, individuals tested and outcomes of testing.

A total of 1,769 people were screened in the study. The mean age of tested participants was 35.68 years (SD 16.1). Of those who were anti-HCV positive, the mean age was 45.4 years (SD 16.3). Most respondents were females, comprising 1,179 (66.7%) of tested individuals. One hundred and forty- nine (149) out of 1,769 participants were anti-HCV positive giving a seroprevalence of 8.4%. Out of 149 individuals who were anti-HCV positive, only 74 were able to undertake HCV RNA testing, because patients must usually pay out-of-pocket for this test. A study flow diagram of testing and results is shown in Fig 1.

**Fig 2:**
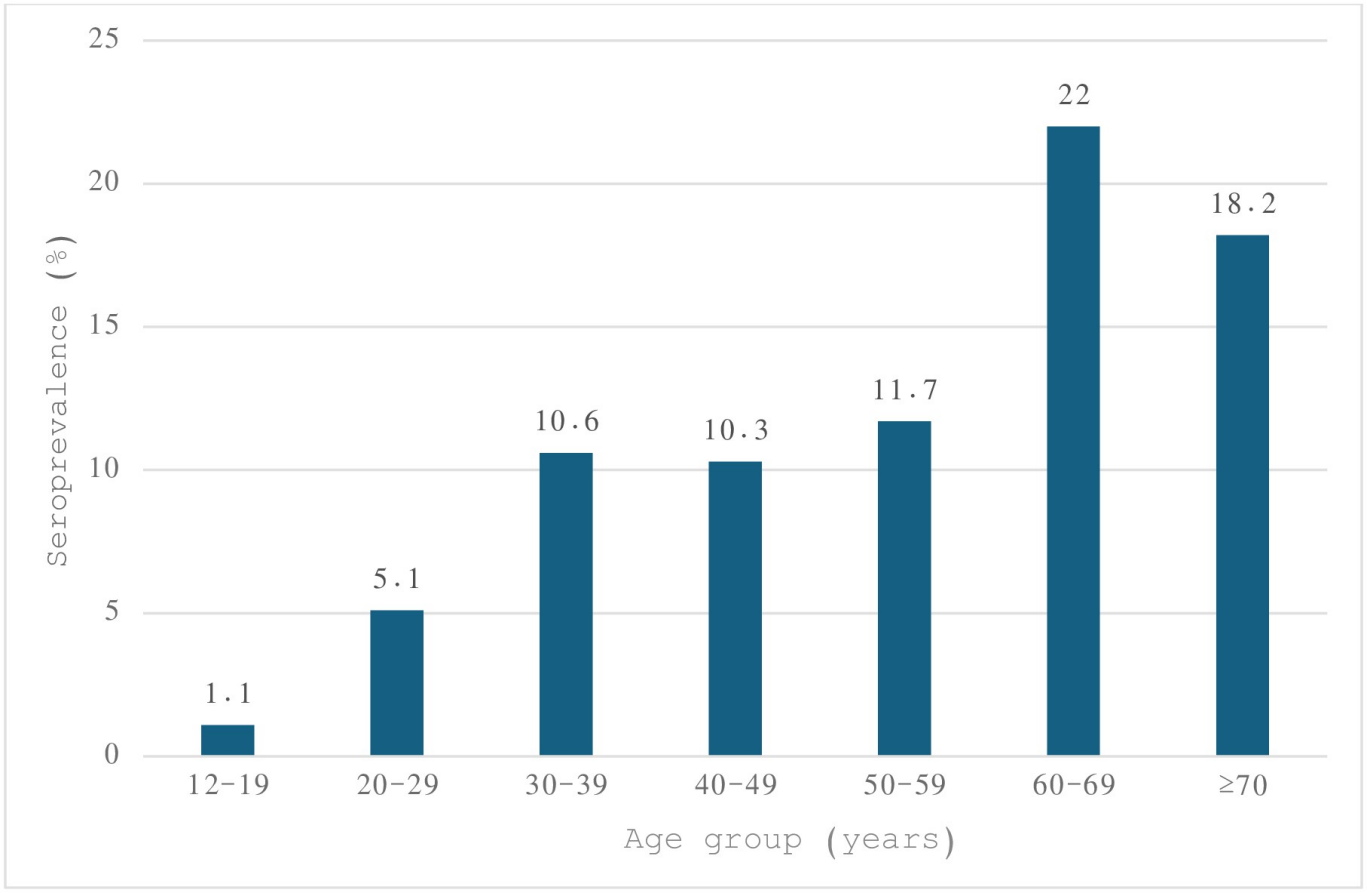
Hepatitis C seroprevalence by age-group based on anti-HCV rapid diagnostic testing. Age groups: 12–19 (n = 350), 20–29 (n = 352), 30-39(n = 360), 40-49(n = 301), 50-59(n = 179), 60-69(n = 82), 70+ (n = 66).

Forty-four (44) out of 74 seropositive cases had viremic infection giving a viremia proportion of 59.5% (Table 1). Thus, the estimated viremic (chronic HCV) prevalence in the screened population was 5.0%. The anti-HCV prevalence increased across the age groups with lowest recorded in the 12–19 age group (1.1%) and highest in those aged 60–69 (22%) (Fig 2).

**Table 1:** Seroprevalence of HCV by demographic information and risk factors.

Variable	Anti-HCV Result		*p*-value*
	Positive, n = 149	Negative, n = 1620	
Age (mean, SD)	45.4 (±16.3)	34.8 (±15.8)	**0.00**
Age Group (years)			
12–19	4 (1.1%)	346 (98.9%)	**0.00**
20–29	18 (5.1%)	334 (94.9%)	
30–39	38 (10.6%)	322 (89.4%)	
40–49	31 (10.3%)	270 (89.7%)	
50–59	21 (11.7%)	158(88.3%)	
60–69	18 (22.0%)	64 (78.0%)	
≥70	12 (18.2%)	54 (81.7%)	
Sex			
Male	57 (9.7%)	531 (90.3%)	0.143
Female	91 (7.7%)	1088 (92.3%)	
History of blood transfusion			
Yes	17 (11.0%)	137 (89.0%)	0.143
No	132 (8.2%)	1483 (91.8%)	
History of surgical/dental procedure			
Yes	35 (12.9%)	237 (87.1%)	**0.003**
No	114 (7.6%)	1383 (92.4%)	
History of tribal / scarification mark			
Yes	79 (8.0%)	909 (92.0%)	0.568
No	70 (9.0%)	711 (91.0%)	
History of FGM / Circumcision			
Yes	79 (12.1)	575	**0.000**
No	71 (6.4)	1042	
History IV drug use			
Yes	12 (12.1%)	87 (87.9%)	0.190
No	137 (8.2%)	1532 (91.8%)	
Accidental prick by a needle/sharp			
Yes	37 (24.8%)	461 (28.5%)	0.293
No	112 (75.2%)	1157 (71.5%)	
History of sexual activity			
Yes	134 (10.2%)	1184 (89.8%)	**0.000**
No	15 (3.3%)	436 (96.7%)	
Positive maternal anti-HCV status			
Don’t Know	93 (8.6%)	988 (91.4%)	**0.000**
No	46 (7.0%)	614 (93.0%)	
Yes	10 (37.0%)	17 (63.0%)	
HIV Infection			
Yes	5 (27.8%)	13 (72.2%)	**0.003**
No	144 (8.2%)	1607 (91.8%)	
HBV Infection			
Yes	3 (7.3%)	38 (92.7%)	0.774
No	146 (8.5%)	1578 (91.5%)	
HCV RNA Result	n/N (%)		
Positive	44/74 (59.5)		
Negative	30/74 (40.5)		

*By t-test or chi square test.

Abbreviations: FGM—female genital mutilation, HIV—human immunodeficiency virus, HBV—hepatitis B virus.

There were significant differences in HCV seropositivity and some risk factor exposures. A higher proportion of individuals with a history of sexual activity (p = 0.000), history of surgical or dental extraction (p = 0.003), female genital mutilation (FGM) or circumcision (p = 0.000), positive maternal history of HCV (p = 0.000) and presence of HIV infection (p = 0.003), were anti-HCV positive compared to those without these exposures (Table 1).

On multivariate logistic regression, increasing age (OR 1.03; 95% Cl 1.01–1.04), positive HIV status (OR 4.03; 95% Cl 1.35–12.05), history of sexual activity (OR 2.57; 95% Cl 1.38–4.79), previous FGM or circumcision (OR 1.63; 95% Cl 1.04–2.55) and positive maternal HCV status (OR 10.38; 95% Cl 4.13–26.05) were found to be predictors of HCV seropositivity (Table 2).

**Table 2:** Multivariate logistic regression analysis of risk factors associated with positive anti-HCV result.

Risk Factor	Adjusted* Odds Ratio	95% CI	p value
Age (years)	1.03	1.01–1.04	**0.000**
Sex			
Male	1.04	0.66–1.65	0.854
Blood transfusion			
Yes	1.10	0.61–2.00	0.751
Surgical or dental procedure			
Yes	1.16	0.75–1.81	0.506
Tribal or Scarification Mark			
Yes	0.85	0.59–1.22	0.378
FGM / Circumcision			
Yes	1.63	1.04–2.55	**0.032**
IV Drug Use			
Yes	1.36	0.66–2.79	0.401
Needlestick injury			
Yes	1.09	0.71–1.67	0.700
Current or previous sexual activity			
Yes	2.57	1.38–4.79	**0.003**
Previous maternal positive test for Anti-HCV			
Yes	10.38	4.13–26.05	**0.000**
HIV Status			
Yes	4.03	1.35–12.05	**0.012**

*Multivariable model adjusted for age (continuous); sex (male, female); all risk factors (yes, no).

## Discussion

In this study, the anti-HCV seroprevalence was 8.4% which was consistent with the seroprevalence found in the northern part of Ghana from a hospital-based study by Nartey et al [10]. Seroprevalence of HCV in Africa is estimated at about 3% [20], and in West Africa. This is estimated at 4.1% with Burkina Faso having one of the highest rates of 6.1% in the West African sub-region [5]. The lowest prevalence in Africa is found in Southern and Eastern Africa, estimated at 0.7% and 3.0% respectively [21,22]. Differences in prevalence may be related to the reliability of the different HCV rapid test kits or serological tests available in the region and the variable population groups screened in previous studies e.g. community vs hospital-based, positive vs negative HIV and HBV status. Some studies have demonstrated the variable performance of anti-HCV RDT kits [23,24]. For example, Waheed et al reported sensitivities and specificities of ranging from 69%–100% and 66%– 100% respectively of various RDTs in Pakistan [23], whilst Jargalsaikhan reported a significantly higher number of false positive anti-HCV results in the presence of HBsAg positivity for different test kits used in Mongolia [24]. To ensure quality standards and high performance, WHO prequalification has been recommended for tests such as anti-HCV RDTs [25]. In Ghana a wide range of test kits are commercially available for anti-HCV screening, many of which are not yet WHO or Ghana FDA pre-qualified [10,26]. Additionally, sociocultural practices e.g. circumcision and tribal scarification, healthcare infrastructure and diagnostic and treatment capabilities may also influence disease burden of HCV in different regions [27]. Furthermore, HCV transmission is increased in high-risk groups including individuals with HIV, those with history of injecting drug use, patients on hemodialysis and those with a history of transplantation. The difference in the burden of risk factors across the continent may also affect the differences in the seroprevalence rates [21].

In this study, the proportion of seropositive persons with viremia was 59.5%, and the estimated viremic (chronic HCV) prevalence in the screened population was therefore estimated to be 5%. Globally, 71.1 million people are viremic giving a prevalence of 1%. It is also estimated that about 10.15 million Africans are viremic [5]. A population-based study demonstrating viremic prevalence in North Africa and the Middle East by Harfouche et al reported a proportion of 67.6% which was similar to this study [28]. The prevalence seen was higher than global viremic prevalence and in a study in Kenya where Mwangi et al found a viremic prevalence of 10% amongst a blood donor population [29,30].

In our study HCV RNA was only available for 74 out of 149 cases. This is due to the high cost of HCV RNA in Ghana, for which patients must pay out-of-pocket. Nartey et al previously reported on the high cost of virological testing, with an average price of RNA of $88.8 [10]. High cost of HCV RNA levels limits further testing for many individuals in sub-Saharan Africa [31]. Sonderup et al highlighted cost-effective methods of detecting viremia after a positive antibody test in the region. HCV core antigen (HCVcAg) test provides a suitable alternative to confirming, monitoring, and ascertaining viremia in clients, and is also less expensive than HCV RNA, with an estimated cost of $20 per test [32]. The sensitivity and specificity for HCV genotypes 1, 2 and 3 can be as high as 93.7% and 98.7% respectively [33]. Studies to validate the performance of HCVcAg in Ghana where the predominant genotypes are 2 (87%) and 1 (13%) [34] may be helpful to provide evidence to policy makers and the government about the utility of this test. Furthermore, the use of pre-existing platforms such as the GeneXpert system used in the diagnosis of tuberculosis could also provide a cheaper alternative to HCV RNA testing [35].

It is therefore recommended that in order to expand access to HCV virological testing and consequently increase the identification of people who require treatment in low resource settings, there is a need to consider cost-effective alternatives or subsidized testing for the general population [5], updating of the diagnostic algorithms to including newer testing strategies such as HCVcAg, and the training of health care personnel, which should all be supported by policy makers and national governments. Examples of how government-supported initiatives have increased HCV diagnosis and treatment are evident in Egypt where more than 50 million of the population were screened and 4 million people treated [36], helping the country to become the first in the world to attain gold tier certification for HCV elimination [37].

In this study, patients who were identified to have viremic infection were linked to a free Hepatitis C treatment project which was launched by the Ghana Health Service in March 2023 following a donation of DAA medication (sofosbuvir-daclatasvir) from the Egyptian government [18]. Prior to the launch of this project, patients with HCV infection had to pay for treatment out-of-pocket, with a 12 week course of treatment averaging $887 [10]. Further studies are needed to determine the impact of this intervention on improving access to treatment in Ghana given the testing barriers associated with HCV in the country.

From our multivariate logistic regression, increasing age (OR– 1.03; 95% Cl 1.01–1.04) was associated with seropositivity. This was consistent with recent studies in Ghana (OR 1.02 95% CI 1.01–1.02) and Burkina Faso (*P* = 0.024) [31,38]. This could be due to the accumulated risk exposure with increasing age. Although our study did not find any relationship between male sex (OR– 1.04; 95% CI 0.66–1.65) and seropositivity, other studies have suggested an increased risk among males (OR 1.26 95% CI 1.08–1.48), (3.9%; 95% CI: 3.4–4.5) in Kenya and Ghana respectively [22,23].

Current or previous sexual activity (P = 0.003) was associated with seropositivity which was consistent with a study by Okafor et al [24] and positive HIV status (P = 0.012) was also associated with seropositivity which was also consistent with work done by Gebrekristos et al in Ethiopia [39], where immunosuppression was associated with anti-HCV seropositivity. Other risk factor associations with seropositivity included previous maternal positive test for anti-HCV (P = 0.000) and history of FGM or circumcision (P = 0.032). Both FGM and male circumcision have previously been identified as significant risk factors for HCV in Egypt, which historically has the highest burden of HCV globally [40,41]. This is associated with the use of unsterilized instruments by traditional FGM operators. In Ghana the Upper East and Upper West regions have the highest rates of FGM and the practice is usually performed by traditional operators [42]. Furthermore, it has been reported that the practice of male circumcision is more likely to be carried out by informal operators among communities that are more rural and that have lower socio-economic status, such as in the northern part of Ghana [43]. For maternal transmission, the Centers for Disease Control (CDC) reports a transmission risk of 6% [44], and further studies are needed in Ghana to determine the perinatal transmission rate in our setting.

Significant factors in previous studies which were contrary to our findings were history of surgical or dental procedure (P = 0.506), tribal or scarification mark (P = 0.378), intravenous drug use (P = 0.401) and needle stick injury. This may be because samples for individuals who had been exposed to these risk factors were few in our studies and further studies among each risk strata can be further investigated.

The HCV-associated risk factors which have been identified in this study can be used to design and implement educational campaigns within the nine communities and more broadly within the Upper East region and may be useful to inform community-based interventions and policies that target prevention of harmful practices that increase the risk of infection.

Study limitations includes high cost and unavailability of HCV RNA testing at the study site and across the country where only 2 major public hospitals run HCV RNA testing, meaning that only about half of those who screened positive undertook virological testing [22]. Another limitation is that although patients with virological testing were linked to the Ghana Health Service free treatment project, follow-up data was not recorded in this study to determine whether they achieved sustained virological response. Furthermore, only persons aged 12 years and above were included in this study due to the enrollment criteria for a free HCV treatment project STOP Hep C Ghana, where children <12 are not yet eligible for the free treatment opportunity [18]. It would be prudent to determine the community-based prevalence in children since this is not yet well described in Ghana. This is needed to inform policy makers on whether this free treatment opportunity should be expanded to include children younger than 12 years, since they are currently excluded from STOP Hep C. The WHO currently recommends treatment with DAA therapy for children aged 3 years and above.

## Conclusion

The anti-HCV prevalence in the Upper East Region of Ghana is high. Almost 60% of individuals who are antibody positive demonstrate viremic infection, however high cost of RNA serves as a barrier to virologic testing, therefore cost-effective diagnostics are needed. There is a need to educate the population about HCV-associated risk factors including circumcision and female genital mutilation, to reduce HCV transmission and burden of disease.
